# Supplementary material for: The interactome of intact mitochondria by cross-linking mass spectrometry provides evidence for coexisting respiratory supercomplexes
Source: Mol Cell Proteomics. 2017 Dec 8;17(2):216–32. doi: 10.1074/mcp.RA117.000470 (PMC5795388; doi:10.1074/mcp.RA117.000470)
Supplement: Supplemental Data [file supp_RA117.000470_133922_0_supp_23978_fzffwf.zip › spectra_annotation/mito_spectra_annotation/38-1-1-1-12-1.pdf]

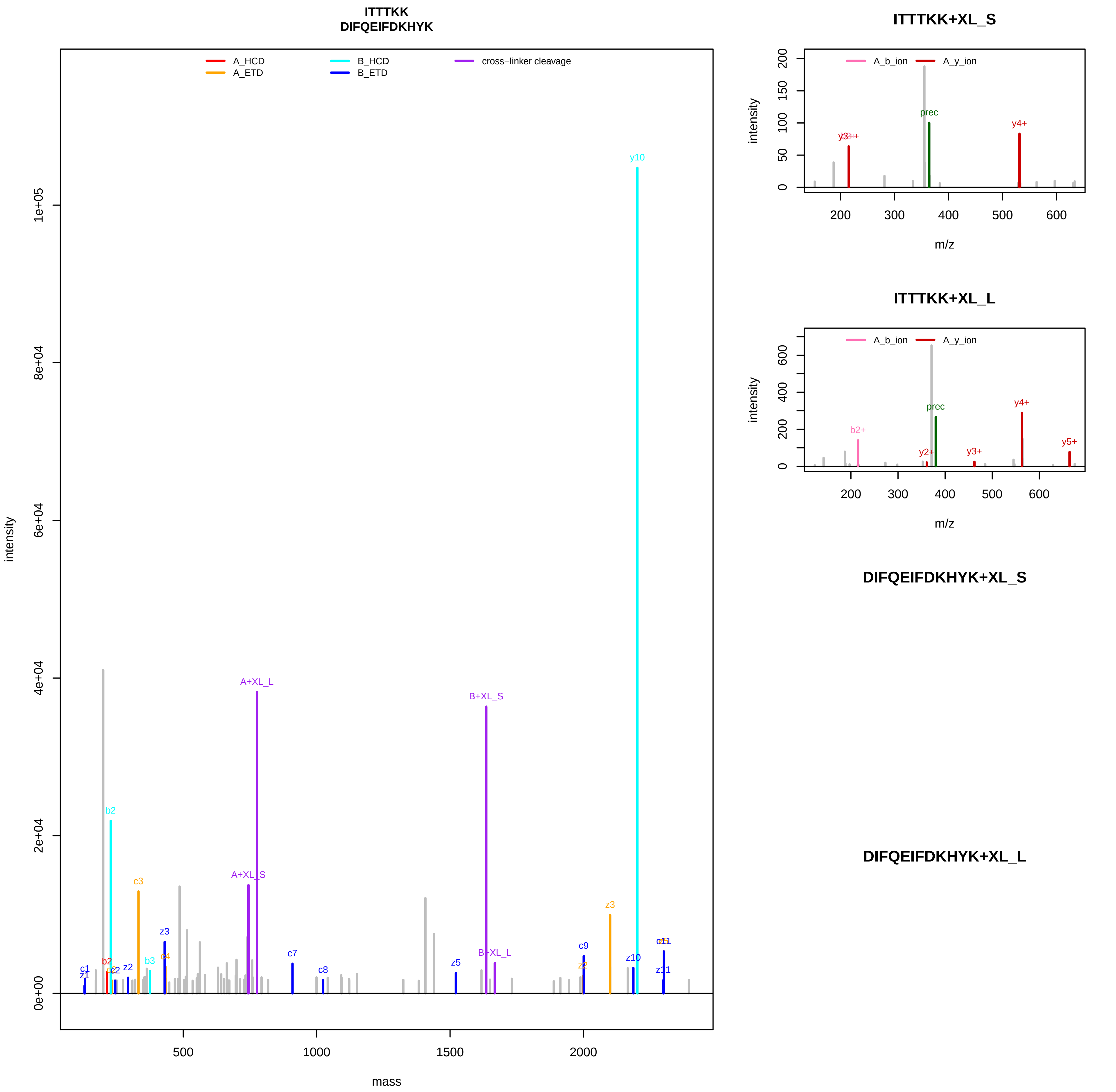
ITTTKK
DIFQEIFDKHYK
A_HCD
A_ETD
B_HCD
B_ETD
cross−linker cleavage
intensity
mass
y10
A+XL_L
B+XL_S
A+XL_S
B+XL_L
b2
b3
c1
z1
c2
z2
c3
z3
c4
c7
c8
z5
c9
z10
c11
z11
0e+00
2e+04
4e+04
6e+04
8e+04
1e+05
500
1000
1500
2000
ITTTKK+XL_S
A_b_ion
A_y_ion
prec
y3++
y4+
m/z
ITTTKK+XL_L
b2+
y2+
y3+
y5+
DIFQEIFDKHYK+XL_S
DIFQEIFDKHYK+XL_L
